# Comparison of the efficacy between intensity-modulated radiotherapy and two-dimensional conventional radiotherapy in stage II nasopharyngeal carcinoma

**DOI:** 10.18632/oncotarget.17481

**Published:** 2017-04-27

**Authors:** Xin-Bin Pan, Kai-Hua Chen, Shi-Ting Huang, Yan-Ming Jiang, Jia-Lin Ma, Zhong-Guo Liang, Song Qu, Ling Li, Long Chen, Xiao-Dong Zhu

**Affiliations:** ^1^ Department of Radiation Oncology, Cancer Hospital of Guangxi Medical University, Nanning, Guangxi, P.R. China

**Keywords:** nasopharyngeal carcinoma, intensity-modulated radiotherapy, two-dimensional conventional radiotherapy

## Abstract

We compared treatment outcomes in patients with stage II nasopharyngeal carcinoma (NPC) treated with intensity-modulated radiotherapy (IMRT) or two-dimensional conventional radiotherapy (2D-CRT). Stage II (2010 UICC/AJCC staging system) NPC patients treated with IMRT (n = 178) or 2D-CRT (n = 73) between January 2007 and December 2014 were retrospectively analyzed. Patients were matched using the propensity score-matching method. The primary endpoint was overall survival (OS). Secondary endpoints were local relapse-free survival (LRFS), regional relapse-free survival (RRFS), distant metastasis-free survival (DMFS), and disease-free survival (DFS). Acute and late toxicity reactions to IMRT and 2D-CRT were also compared. In an unmatched cohort of 251 patients, no significant survival differences were found between those receiving IMRT and those receiving 2D-CRT (5-year OS, 95.67% *vs* 94.44%, *P* = 0.0556; LRFS, 97.34% *vs* 98.59%, *P* = 0.6656; RRFS, 99.26% *vs* 100%, *P* = 0.6785; DMFS, 96.5% *vs* 98.63%, *P* = 0.7910; DFS, 92.2% *vs* 97.24%, *P* = 0.8719). In the propensity-matched cohort of 146 patients, 5-year OS (97.06% *vs* 94.44%, *P* = 0.1325), LRFS (96.75% *vs* 98.59%, *P* = 0.8869), RRFS (100% *vs* 100%, *P* = 1.0000), DMFS (98.63% *vs* 98.63%, *P* = 0.4225), and DFS (95.37% *vs* 97.24%, *P* = 0.5634) were similar between patients treated with IMRT or 2D-CRT. However, IMRT correlated with fewer acute and late toxicity reactions. Thus although IMRT provides no survival advantage, it has a lower incidence of toxicity than 2D-CRT in stage II NPC patients.

## INTRODUCTION

Nasopharyngeal carcinoma (NPC) is an endemic disease in Southern China. Radiotherapy is the primary treatment modality for NPC, and two-dimensional conventional radiotherapy (2D-CRT) is effective in the control of NPC. However, complications of 2D-CRT are severe and lifelong. Therefore, 2D-CRT has been widely replaced by intensity-modulated radiotherapy (IMRT) because of the technical and dosimetric superiority of IMRT. IMRT is the preferred radiation technique when resources permit.

IMRT is expected to improve patient survival and reduce toxicity, but superiority of IMRT over 2D-CRT is not conclusively proved. A meta-analysis [1] suggested that IMRT is correlated with better 5-year overall survival (OS) and local relapse-free survival (LRFS) and a lower incidence of late toxicities. However, two systematic reviews [2, 3] showed that xerostomia was minimally improved by IMRT without improvement in OS and LRFS. In a subgroup analysis, a retrospective study [4] with long-term follow-up reported that IMRT improved OS in stage II NPC patients, but IMRT was reported in other studies to provide improved OS in stage III NPC patients [5] and not in stage II NPC patients [5, 6].

The reported incidence of stage II NPC has increased because of improvements in diagnosis. However, many patients are still treated with 2D-CRT rather than IMRT because of limited access to IMRT and the cost of IMRT in developing countries. This study was conducted to determine whether 2D-CRT is a reasonable treatment option for stage II NPC patients compared with IMRT. The result of this study might help clinicians make treatment decisions, especially in limited-resource settings.

## RESULTS

### Patients

A total of 251 stage II NPC patients were included. Among these patients, 178 were treated with IMRT, 73 patients were treated with 2D-CRT, 94 received radiotherapy alone, 103 received concurrent chemoradiotherapy, and 54 received concurrent chemoradiotherapy with adjuvant chemotherapy. Table 1 summarizes patient characteristics.

**Table 1:** Characteristics of IMRT and 2D-CRT patients in the unmatched cohort and the propensity-matched cohort

	The unmatched cohort				The propensity-matched cohort			
	IMRT (n = 178)	2D-CRT (n = 73)	Total (n = 251)	P	IMRT (n = 73)	2D-CRT (n = 73)	Total (n = 146)	P
Age				0.1376				0.8347
Mean±SD	44.48±9.11	46.45±10.44	45.06±9.53		46.10±10.15	46.45±10.44	46.27±10.26	
Median	43	44	44		45	44	44	
Range	22–69	31–68	22–69		22–69	31–68	22–69	
Sex				0.5447				0.8609
Female	54 (30.34%)	25 (34.25%)	79 (31.47%)		24 (32.88%)	25 (34.25%)	49 (33.56%)	
Male	124 (69.66%)	48 (65.75%)	172 (68.53%)		49 (67.12%)	48 (65.75%)	97 (66.44%)	
Pathology				0.0380				0.0431
WHO II	23 (12.92%)	3 (4.11%)	26 (10.36%)		10 (13.70%)	3 (4.11%)	13 (8.90%)	
WHO III	155 (87.08%)	70 (95.89%)	225 (89.64%)		63 (86.30%)	70 (95.89%)	133 (91.10%)	
T-stage				0.1588				0.6821
T1	26 (14.61%)	16 (21.92%)	42 (16.73%)		14 (19.18%)	16 (21.92%)	30 (20.55%)	
T2	152 (85.39%)	57 (78.08%)	209 (83.27%)		59 (80.82%)	57 (78.08%)	116 (79.45%)	
N-stage				0.0012				0.5990
N0	30 (16.85%)	26 (35.62%)	56 (22.31%)		23 (31.51%)	26 (35.62%)	49 (33.56%)	
N1	148 (83.15%)	47 (64.38%)	195 (77.69%)		50 (68.49%)	47 (64.38%)	97 (66.44%)	
Clinical stage				0.0010				0.7082
T1N1M0	30 (16.85%)	16 (21.92%)	46 (18.33%)		14 (19.18%)	16 (21.92%)	30 (20.55%)	
T2N0M0	30 (16.85%)	26 (35.62%)	56 (22.31%)		23 (31.51%)	26 (35.62%)	49 (33.56%)	
T2N1M0	118 (66.29%)	31 (42.47%)	149 (59.36%)		36 (49.32%)	31 (42.47%)	67 (45.89%)	
Treatment				<0.0001				0.4175
RT	51 (28.65%)	43 (58.90%)	94 (37.45%)		50 (68.49%)	43 (58.90%)	93 (63.70%)	
CCRT	87 (48.88%)	16 (21.92%)	103 (41.04%)		14 (19.18%)	16 (21.92%)	30 (20.55%)	
CCRT+AC	40 (22.47%)	14 (19.18%)	54 (21.51%)		9 (12.33%)	14 (19.18%)	23 (15.75%)	

IMRT: intensity-modulated radiotherapy.

2D-CRT: two-dimensional conventional radiotherapy.

SD: standard deviation.

RT: radiotherapy.

CCRT: concurrent chemoradiotherapy.

AC: adjuvant chemotherapy.

### Follow-up

The endpoint of the follow-up was October 19, 2016. Median follow-up time was 40 months (12-110 months) in the IMRT group and 73.5 months (31-1-16 months) in the 2D-CRT group. The follow-up rate was 96.81%, with 8 patients lost to follow-up. The details of the treatment failure pattern are shown in Table 2.

**Table 2:** Patterns of treatment failure

	IMRT (*n* = 178)	2D-CRT (*n* = 73)
Death	5	6
Local relapse	6	3
Regional relapse	1	0
Distant metastasis	5	1

IMRT: intensity-modulated radiotherapy.

2D-CRT: two-dimensional conventional radiotherapy.

Salvage treatment was received by 12 patients. Among those patients, 6 with local relapse received radiotherapy plus chemotherapy, 1 with local relapse received nasopharyngectomy, 1 with regional relapse received neck dissection, and 4 with distant metastasis received chemotherapy. Traditional Chinese medicine was received by 4 patients.

### Survival outcomes

**Figure 1:**
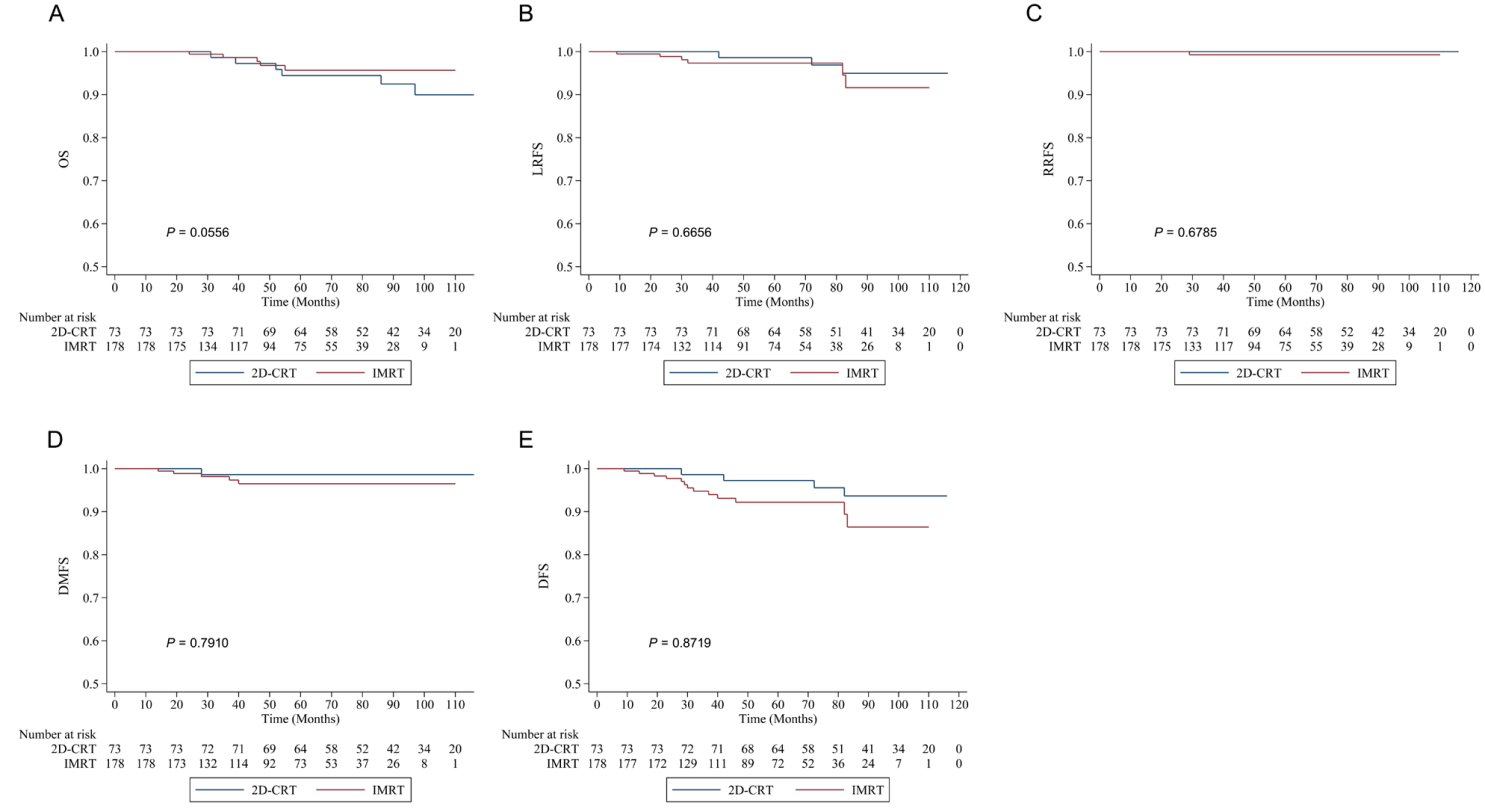
Kaplan-Meier survival curves of intensity-modulated radiotherapy (IMRT) *versus* two-dimensional conventional radiotherapy (2D-CRT) in the unmatched cohort **A.** Overall survival (OS). **B.** Local relapse-free survival (LRFS). **C.** Regional relapse-free survival (RRFS). **D.** Distant metastasis-free survival (DMFS). **E.** Disease-free survival (DFS).

In the unmatched cohort, IMRT did not improve OS, LRFS, RRFS, DMFS, or DFS (5-year OS 95.67% *vs* 94.44%, *P* = 0.0556; LRFS 97.34% *vs* 98.59%, *P* = 0.6656; RRFS 99.26% *vs* 100%, *P* = 0.6785; DMFS 96.5% *vs* 98.63%, *P* = 0.7910; DFS 92.20% *vs* 97.24%, *P* = 0.8719) compared with 2D-CRT (Figure 1).

**Figure 2:**
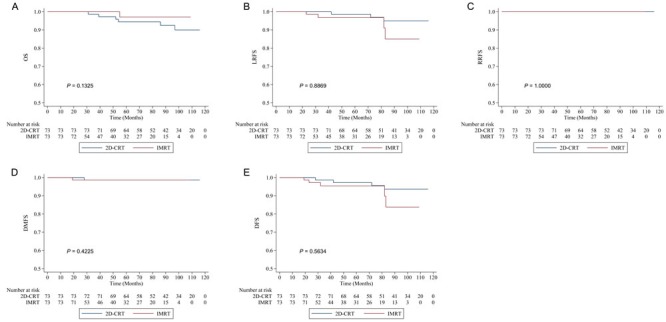
Kaplan-Meier survival curves of intensity-modulated radiotherapy (IMRT) *versus* two-dimensional conventional radiotherapy (2D-CRT) in the propensity-matched cohort **A.** Overall survival (OS). **B.** Local relapse-free survival (LRFS). **C.** Regional relapse-free survival (RRFS). **D.** Distant metastasis-free survival (DMFS). **E.** Disease-free survival (DFS).

In the propensity-matched cohort, IMRT-treated patients showed similar survival to those treated with 2D-CRT (5-year OS 97.06% *vs* 94.44%, *P* = 0.1325; LRFS 96.75% *vs* 98.59%, *P* = 0.8869; RRFS 100% *vs* 100%, *P* = 1.0; DMFS 98.63% *vs* 98.63%, *P* = 0.4225; DFS 95.37% *vs* 97.24%, *P* = 0.5634) (Figure 2).

Adjusting for prognostic factors, IMRT showed similar efficacy to 2D-CRT in management of death, local relapse, regional relapse, and distant metastasis, in both the unmatched cohort and the propensity-matched cohort. The result of the multivariate analysis is showed in Table 3.

**Table 3:** Subgroup analysis of IMRT versus 2DCRT in multivariate analysis

	Subgroup	The unmatched cohort		The propensity-matched cohort	
		HR (95% CI)	*P*	HR (95% CI)	*P*
OS	2D-CRT *vs* IMRT	1.5742 (0.4668, 5.3083)	0.4644	2.9179 (0.3456, 24.6346)	0.3252
LRFS	2D-CRT *vs* IMRT	0.3626 (0.0781, 1.6838)	0.1954	0.3233 (0.0685, 1.5251)	0.1537
RRFS	2D-CRT *vs* IMRT	N/A	N/A	N/A	N/A
DMFS	2D-CRT *vs* IMRT	0.3899 (0.0454, 3.3490)	0.3906	0.8680 (0.0539, 13.9739)	0.9205
DFS	2D-CRT *vs* IMRT	0.3450 (0.1057, 1.1262)	0.0779	0.3924 (0.1002, 1.5375)	0.1794

IMRT: intensity-modulated radiotherapy.

2D-CRT: two-dimensional conventional radiotherapy

OS: overall survival.

LRFS: local relapse-free survival.

RRFS: regional relapse-free survival.

DMFS: distant metastasis-free survival.

DFS: disease-free survival.

N/A: not applicable.

HR: hazard ratio.

### Toxicity reactions

No grade 4 acute or late toxicity reactions were found in any patients, but grade 3 toxicity reactions were observed in some patients. Acute toxicity reactions such as greater mucositis and skin reaction were correlated with 2D-CRT in the propensity-matched cohort but not in the unmatched cohort. Late toxicity reactions such as deafness/otitis, skin fibrosis, trismus, and xerostomia were correlated with IMRT in both the propensity-matched cohort and the unmatched cohort. The details of acute and late toxicity reactions are shown in Table 4.

**Table 4:** Toxicity reactions to IMRT and 2D-CRT in the unmatched cohort and the propensity-matched cohort

	The unmatched cohort				The propensity-matched cohort			
	IMRT (*n* =178)	2D-CRT (*n* = 73)	Total (*n* = 251)	*P*	IMRT (*n* = 73)	2D-CRT (*n* = 73)	Total (*n* = 146)	*P*
**Acute toxicity reactions**								
Leukopenia				<0.0001				0.0001
1	45 (25.28%)	6 (8.22%)	51 (20.32%)		21 (28.77%)	6 (8.22%)	27 (18.49%)	
2	53 (29.78%)	4 (5.48%)	57 (22.71%)		12 (16.44%)	4 (5.48%)	16 (10.96%)	
3	24 (13.48%)	2 (2.74%)	26 (10.36%)		2 (2.74%)	2 (2.74%)	4 (2.74%)	
Leukopenia				<0.0001				0.4694
1	37 (20.79%)	6 (8.22%)	43 (17.13%)		5 (6.85%)	6 (8.22%)	11 (7.53%)	
2	36 (20.22%)	5 (6.85%)	41 (16.33%)		8 (10.96%)	5 (6.85%)	13 (8.90%)	
3	9 (5.06%)	1 (1.37%)	10 (3.98%)		2 (2.74%)	1 (1.37%)	3 (2.05%)	
Anemia				<0.0001				0.0315
1	39 (21.91%)	4 (5.48%)	43 (17.13%)		8 (10.96%)	4 (5.48%)	12 (8.22%)	
2	19 (10.67%)	2 (2.74%)	21 (8.37%)		6 (8.22%)	2 (2.74%)	8 (5.48%)	
3	2 (1.12%)	0 (0%)	2 (0.80%)		1 (1.37%)	0 (0%)	1 (0.68%)	
Thrombocytopenia				0.1107				0.4716
1	11 (6.18%)	3 (4.11%)	14 (5.58%)		5 (6.85%)	3 (4.11%)	8 (5.48%)	
2	4 (2.25%)	0 (0%)	4 (1.59%)					
3	3 (1.69%)	0 (0%)	3 (1.20%)					
Liver dysfunction				0.0170				0.4205
1	32 (17.98%)	6 (8.22%)	38 (15.14%)		7 (9.59%)	6 (8.22%)	13 (8.90%)	
2	7 (3.93%)	1 (1.37%)	8 (3.19%)		3 (4.11%)	1 (1.37%)	4 (2.74%)	
3	1 (0.56%)	0 (0%)	1 (0.40%)					
Renal dysfunction				0.1660				0.6491
1	13 (7.30%)	2 (2.74%)	15 (5.98%)		3 (4.11%)	2 (2.74%)	5 (3.42%)	
Nausea/vomiting				<0.0001				0.2648
1	35 (19.66%)	14 (19.18%)	49 (19.52%)		16 (21.92%)	14 (19.18%)	30 (20.55%)	
2	84 (47.19%)	15 (20.55%)	99 (39.44%)		21 (28.77%)	15 (20.55%)	36 (24.66%)	
3	10 (5.62%)	2 (2.74%)	12 (4.78%)		1 (1.37%)	2 (2.74%)	3 (2.05%)	
Weight loss				0.0174				0.8822
1	64 (35.96%)	31 (42.47%)	95 (37.85%)		17 (23.29%)	31 (42.47%)	48 (32.88%)	
2	48 (26.97%)	8 (10.96%)	56 (22.31%)		15 (20.55%)	8 (10.96%)	23 (15.75%)	
3	1 (0.56%)	0 (0%)	1 (0.40%)		1 (1.37%)	0 (0%)	1 (0.68%)	
Mucositis				0.7069				0.0482
1	11 (6.18%)	2 (2.74%)	13 (5.18%)		9 (12.33%)	2 (2.74%)	11 (7.53%)	
2	121 (67.98%)	56 (76.71%)	177 (70.52%)		54 (73.97%)	56 (76.71%)	110 (75.34%)	
3	46 (25.84%)	15 (20.55%)	61 (24.30%)		10 (13.70%)	15 (20.55%)	25 (17.12%)	
Skin reaction				0.7600				0.0342
1	34 (19.10%)	10 (13.70%)	44 (17.53%)		18 (24.66%)	10 (13.70%)	28 (19.18%)	
2	112 (62.92%)	52 (71.23%)	164 (65.34%)		50 (68.49%)	52 (71.23%)	102 (69.86%)	
3	32 (17.98%)	11 (15.07%)	43 (17.13%)		5 (6.85%)	11 (15.07%)	16 (10.96%)	
**Late toxicity reactions**								
Deafness/otitis				0.0003				<0.0001
1	76 (44.19%)	17 (23.94%)	93 (38.27%)		38 (53.52%)	17 (23.94%)	55 (38.73%)	
2	79 (45.93%)	53 (74.65%)	132 (54.32%)		24 (33.80%)	53 (74.65%)	77 (54.23%)	
3	6 (3.49%)	1 (1.41%)	7 (2.88%)		1 (1.41%)	1 (1.41%)	2 (1.41%)	
Skin fibrosis				<0.0001				<0.0001
1	132 (76.74%)	3 (4.23%)	135 (55.56%)		55 (77.46%)	3 (4.23%)	58 (40.85%)	
2	30 (17.44%)	62 (87.32%)	92 (37.86%)		10 (14.08%)	62 (87.32%)	72 (50.70%)	
3	1 (0.58%)	6 (8.45%)	7 (2.88%)		0 (0%)	6 (8.45%)	6 (4.23%)	
Trismus				<0.0001				<0.0001
1	146 (84.88%)	18 (25.35%)	164 (67.49%)		60 (84.51%)	18 (25.35%)	78 (54.93%)	
2	12 (6.98%)	51 (71.83%)	63 (25.93%)		3 (4.23%)	51 (71.83%)	54 (38.03%)	
3	1 (0.58%)	2 (2.82%)	3 (1.23%)		0 (0%)	2 (2.82%)	2 (1.41%)	
Xerostomia				<0.0001				<0.0001
1	133 (77.33%)	3 (4.23%)	136 (55.97%)		51 (71.83%)	3 (4.23%)	54 (38.03%)	
2	28 (16.28%)	49 (69.01%)	77 (31.69%)		13 (18.31%)	49 (69.01%)	62 (43.66%)	
3	3 (1.74%)	19 (26.76%)	22 (9.05%)		0 (0%)	19 (26.76%)	19 (13.38%)	

IMRT: intensity-modulated radiotherapy.

2D-CRT: two-dimensional conventional radiotherapy.

## DISCUSSION

The study suggests that IMRT provided no survival advantage compared with 2D-CRT in stage II NPC patients, but IMRT was correlated with fewer acute and late toxicity reactions. If resources do not permit IMRT, 2D-CRT is a reasonable treatment option for stage II NPC patients. The result of this study might aid clinicians in making treatment decisions, especially in limited-resource settings.

The benefit of IMRT results from two dosimetric advantages: (1) improved conformity of target dose and facilitation of dose escalation and (2) decreased radiation dose to organs at risk, thus reducing treatment toxicities [7–12]. Studies that utilized IMRT to treat NPC patients reported increased LRFS and less late toxicity compared with 2D-CRT [13–16]. IMRT is recommended for NPC patients by the NCCN guidelines. However, two systematic reviews [2, 3] showed that only xerostomia was minimally improved by IMRT without improvement of OS and LRFS. Whether the dosimetric advantages of IMRT translate into clinical benefit is not clear. Moreover, the clinical stage of the patients who would benefit from IMRT is also unclear [4–6].

The reported incidence of stage II NPC has greatly increased because of improvements in diagnosis. However, many patients in developing regions can only receive 2D-CRT. The cost of IMRT is a problem for many patients [17, 18]. Clinicians urgently need a recommendation to make treatment decisions for stage II NPC patients. However, previous studies categorized NPC patients as a single group and analyzed survival outcomes [4–6, 14]. Most previous studies were retrospective analyses [4, 6, 14]. The prospective, randomized study mainly focused on stage III and stage IV patients [5]. The contradictory results of these studies did not provide accurate information regarding the efficacy of IMRT *versus* 2D-CRT. The best radiotherapy technology for stage II NPC patients is still the subject of controversy. Prospectively comparing the outcomes of IMRT and 2D-CRT in NPC patients in a randomized controlled trial is impractical. Thus, the present study used the PSM method to create similar IMRT and 2D-CRT groups to reduce possible biases [19]. Moreover, data of this study were collected from a single institution in an endemic area where clinicians had expertise in diagnosing and treating NPC.

IMRT is expected to improve LRFS and RRFS in advanced loco-regional NPC through escalation of the applied radiation dose to the tumor [13–16]. Both physical dose escalation and accelerated fractionation could result in the greatest tumor kill rate. However, improvement of LRFS and RRFS by IMRT was not significant in stage II NPC patients in our study. A possible reason for this outcome was that 2D-CRT plus a 70-Gy boost therapy dose provided excellent loco-regional control. The similar local-regional control rates of IMRT and 2D-CRT might produce similar DMFS and OS.

A recent study [6] used the PSM method to retrospectively assess the survival differences between patients who received IMRT and patients who received 2D-CRT in a large cohort of NPC patients receiving radiotherapy alone. The study showed that IMRT had no advantage over 2D-CRT in OS, LRFS, or DMFS, irrespective of T-stage, N-stage, or clinical stage. In clinical practice, it is impractical to treat stage II NPC patients without chemotherapy. Moreover, the study [6] did not report acute and late toxicities with IMRT and 2D-CRT. The present study suggested a similar result but a lower incidence of toxicities than with 2D-CRT in stage II NPC patients, with or without chemotherapy. The result of the present study might provide more information to clinicians and NPC patients, especially in developing regions.

The major advantage of this study is the use of PSM. Acute toxicity mucositis and skin reactions were correlated with 2D-CRT as in the unmatched cohort. The incidence of grade 2 to 3 mucositis and skin reaction was higher in the IMRT group than in the 2D-CRT group in the propensity-matched cohort. Use of the PSM can reduce the biases caused by confounding variables.

This study had the following limitations: (1) Patients who received 2D-CRT had a longer follow-up time, because more 2D-CRT was administered during the first few years. However, IMRT was administered more during recent years. Patients who received IMRT had a relatively shorter follow-up time. The follow-up time might be insufficient for observing the survival outcome in the IMRT group.(2) Only 73 patients who received 2D-CRT were included in this study. Moreover, the risk of treatment failure was very low in stage II NPC patients. These aspects might reduce the statistical power. For further study, a longer follow-up time and a larger sample of patients are necessary to verify the results.

In conclusion, this study suggests that IMRT has no survival advantage over 2D-CRT in stage II NPC patients, and 2D-CRT is a reasonable treatment option for stage II NPC patients compared with IMRT in limited-resource settings. IMRT is recommended if resources permit.

## MATERIALS AND METHODS

### Patients

Untreated NPC patients in the Cancer Hospital of Guangxi Medical University were retrospectively analyzed from January 2007 to December 2014. All patients had complete pretreatment evaluations, including a thorough history, a physical examination, hematology, a biochemical profile, electrocardiography, nasopharyngoscopy with biopsy, magnetic resonance imaging (MRI) or computed tomography (CT) scan of the nasopharynx and neck, chest radiography or CT scan, abdominal sonography or CT scan, and whole-body bone scan. Patients were restaged in accordance with the 2010 International Union Against Cancer/American Joint Committee on Cancer (UICC/AJCC) staging system [20]. Patients with stage II NPC were included.

### Radiotherapy

Patients received 2D-CRT in two phases. In the first phase, patients were irradiated by 6-megavolt bilateral and opposing photon beams. The dose for the faciocervical field and the lower anterior cervical field was 36 Gy. In the second phase, the dose for primary tumors was increased from 66 Gy to 70 Gy. The prescribed radiation dose was 2 Gy per fraction, with 5 daily fractions per week.

Patients received IMRT per the International Commission on Radiation Units and Measurements Report 62 guidelines. Gross tumor volumes of nasopharynx (GTVnx) and gross tumor volumes of cervical lymph nodes (GTVnd) were shown by CT/MRI scans. Clinical target volume (CTV) included the GTV with a 1-cm margin, the entire nasopharyngeal space, and the positive lymph node regions. The prescribed radiation doses were 66 Gy to 70.06 Gy in 30 to 31 fractions for GTV and 54 Gy to 60 Gy in 30 fractions for CTV, with 5 daily fractions per week.

### Chemotherapy

Concurrent chemotherapy was 80 to 100 mg/m^2^ of cisplatin for 1 or 3 days in a cycle on days 1, 22, and 43 during radiotherapy. Chemotherapy was postponed or discontinued for patients who experienced serious toxicity and could not recover before the next scheduled radiation treatment. Adjuvant chemotherapy was 80 to 100 mg/m^2^ of cisplatin for 1 day or 3 days, 600-750 mg/m^2^/d of 5-fluorouracil in continuous intravenous infusion for 96 hours or 120 hours in a cycle of 28 days for 2 to 3 cycles.

### Follow-up

Patients were followed up every 3 months through the first 2 years, every 6 months for the next 3 years, and then annually. Physical examination, nasopharyngoscopy, chest radiography or CT scan; abdominal sonography or CT; and MRI or CT scan of the nasopharynx and neck were performed. Bone scan was conducted if clinically indicated.

### Endpoints and patient assessment

The primary endpoint was OS. Secondary endpoints were LRFS, regional relapse-free survival (RRFS), distant metastasis-free survival (DMFS), and disease-free survival (DFS). OS, LRFS, RRFS, and DMFS were defined as the duration from the first day of treatment to the time of death, nasopharyngeal relapse, regional lymph node relapse, or distant metastasis. DFS was the duration from the date of treatment to the date of relapse, metastasis, or death by any cause.

Acute toxicities were assessed by use of the Common Terminology Criteria for Adverse Events (CTCAE, V 4.0). Late toxicities were evaluated according to the toxicity criteria of the Radiation Therapy Oncology Group (RTOG) at each follow-up.

Salvage treatments were given to patients after documented relapse or when the disease was persistent. The salvage treatments included re-irradiation, chemotherapy, and surgery.

### Statistical analysis

Continuous data were analyzed by Student's *t*-test and were expressed as the median ± standard deviation, and categorical variables were analyzed by the χ^2^ test or Fisher's exact test and were presented as percentages. The Kaplan-Meier method was used to calculate survival rates. The log-rank test was used to assess differences between survival curves. Cox proportional hazards regression was performed for univariate and multivariate analysis. Variables with values of *P* < 0.05 in the univariate analysis were further included in the multivariate Cox proportional hazards regression analysis.

Based on the propensity score-matching (PSM) method, one-to-one nearest-neighbor matching without replacement was adopted to overcome selection bias in both groups by use of a 0.1 caliper. The propensity score calculated by a logistic regression model represents the probability of each patient being assigned to IMRT or 2D-CRT. Variables that are possible factors in survival rates were used in the PSM, including age, sex, histology (WHO II, differentiated non-keratinizing carcinoma; WHO III, undifferentiated non-keratinizing carcinoma), T-stage, N-stage, clinical stage, and treatment modality.

Statistical analyses were performed by IBM SPSS Statistics Version 18.0 (IBM Co., Armonk, NY, USA) and the STATA Version 13.0 (StataCorp, College Station, Texas, USA). Two-tailed *P* < 0.05 values were considered statistically significant.

### Ethical statement

This study was approved by the Ethics Committee of Cancer Hospital of Guangxi Medical University.

## Abbreviations

NPC: nasopharyngeal carcinoma
IMRT: intensity-modulated radiotherapy
2D-CRT: two-dimensional conventional radiotherapy
OS: overall survival
LRFS: local relapse-free survival
RRFS: regional relapse-free survival
DMFS: distant metastasis-free survival
DFS: disease-free survival
PSM: propensity score-matching
